# Impedance spectroscopy of single bacterial nanofilament reveals water-mediated charge transfer

**DOI:** 10.1371/journal.pone.0191289

**Published:** 2018-01-19

**Authors:** Artem Grebenko, Vyacheslav Dremov, Petr Barzilovich, Anton Bubis, Konstantin Sidoruk, Tatiyana Voeikova, Zarina Gagkaeva, Timur Chernov, Evgeny Korostylev, Boris Gorshunov, Konstantin Motovilov

**Affiliations:** 1 Moscow Institute of Physics and Technology, Institute lane 9, Dolgoprudny, Russian Federation; 2 Institute of Solid State Physics (RAS), Academician Osipyana street 2, Chernogolovka, Russia; 3 Institute of Problems of Chemical Physics (RAS), Academician Semenov avenue 1, Chernogolovka, Russia; 4 Scientific Center of Russian Federation Research Institute for Genetics and Selection of Industrial Microorganisms, 1-st Dorozhniy pr., 1, Moscow, Russia; US Naval Research Laboratory, UNITED STATES

## Abstract

For decades respiratory chain and photosystems were the main firing field of the studies devoted to mechanisms of electron transfer in proteins. The concept of conjugated lateral electron and transverse proton transport during cellular respiration and photosynthesis, which was formulated in the beginning of 1960-s, has been confirmed by thousands of experiments. However, charge transfer in recently discovered bacterial nanofilaments produced by various electrogenic bacteria is regarded currently outside of electron and proton conjugation concept. Here we report the new study of charge transfer within nanofilaments produced by *Shewanella oneidensis* MR-1 conducted in atmosphere of different relative humidity (RH). We utilize impedance spectroscopy and DC (direct current) transport measurements to find out the peculiarities of conductivity and Raman spectroscopy to analyze the nanofilaments’ composition. Data analysis demonstrates that apparent conductivity of nanofilaments has crucial sensitivity to humidity and contains several components including one with unusual behavior which we assign to electron transport. We demonstrate that in the case of *Shewanella oneidensis* MR-1 charge transfer within these objects is strongly mediated by water. Basing on current data analysis of conductivity we conclude that the studied filaments of *Shewanella oneidensis* MR-1 are capable of hybrid (conjugated) electron and ion conductivity.

## Introduction

According to the recent findings, electron efflux from cytosol in various electrogenic microorganisms (*Geobacter sulfurreducens* [1, 2], *Shewanella oneidensis* [1, 3], *etc.*) occurs via nanofilaments. The structure of these bacterial organelles is not universal for different species. Conductive filaments in *S. oneidensis* MR-1 are supposed to be the outgrowths of the outer cellular membrane [4, 5]. In the case of *G. sulfurreducens* [6] the nanowires are type IVa pili. According to current data the composition of nanofilaments depends crucially not only on particular species but also on cultivation conditions [7, 8]. Notwithstanding the significant progress in crystallization of various multiheme cytochromes of *Shewanella* [9] and recent success in electron microscopy studies [4, 5], the structural organization of conductive nanofilaments remains questionable.

The most intriguing results on conductivity of *S. oneidensis* nanofilaments were obtained by Gorby *et al.* [3] They demonstrated that nanowires are essentially conductive objects. The same group fabricated a field-effect transistor on a single bacterial nanowire [10]. Investigating the dependence of the nanofilament conductance on gate voltage they proved that the charge carrier sign is positive. Probably the most traditional method to determine and analyze the charge transfer mechanism is impedance spectroscopy (or dielectric spectroscopy) in a wide range of frequencies and temperatures. It includes optical measurements at frequencies above MHz and transport measurements at lower frequencies.

Up to now all reported attempts to quantify conductivity of individual *Shewanella oneidensis* MR-1 nanofilament were based on DC measurements [3, 10–12] at room temperature. At the same time there was interesting report [13] aimed to demonstrate peculiarities of charge transfer in *Geobacter sulfurreducens* biofilms depending on humidity and temperature in comparison with classic osmium-based redox-conductor, doped polyaniline and *Shewanella oneidensis* MR-1 biofilm. This study, like several others of the recent years [14–17], oppose to the concept of organometallic conductivity within pili of *Geobacter sulfurreducens* supported by the group of Lovley [18–21]. However, the most recent article devoted to the thorough study of electron transfer in isolated *Geobacter sulfurreducens* pili under physiologically relevant conditions again speak in favor of coherent electron transfer [22]. However, the most recent attempt to model electron transfer in pili of *Geobacter sulfurreducens* by means of DFT with NEGF technique [23] based on the NMR-obtained structure of pilin protein [6] speak in favor of incoherent electron transfer at low voltage biases.

Nevertheless the last decade provided the series of interesting observations of conductivity mechanisms in artificial peptide and protein self-assembling systems [24–27] and melanin [28, 29]. Besides wide structural discrepancies of the objects, which were not limited by the standard amino acids, the range of applied experimental techniques was also more diverse than in the studies of bacterial filaments mentioned above. Humidity control and dependence of conductivity on humidity were tightly studied. Application of aforementioned impedance spectroscopy technique in combination with transient current measurements [26] in the case of peptides and heavy water probing combined with EPR and muon spin relaxation analysis [28, 30] in the case of melanin,revealed the presence of electron and proton contributions in the observed conductivities.

Here we report our study performed by means of impedance spectroscopy of *S. oneidensis* individual filaments. The measurements were carried out at a given carefully controlled values of relative humidity. We demonstrate that conductivity of the filaments has several contributions with quite different dependence on humidity. We associate the most sustained contribution with electrons(holes) as charge carriers. The presence of multiheme cytochromes in the studied filaments was validated by means of Raman spectroscopy.

## Materials and methods

We report about the study of the effect of hydration on charge transfer in nanofilaments of *Shewanella oneidensis* MR-1. We skipped the processes of chemical fixation (in course of extraction) and drying after the immobilization, due to our concerns on the integrity of electroactive parts of material. In the study we used either individual nanowires or the groups of spatially separated nanowires completely detached from bacterial bodies. The procedure of cultivation was described in details in Motovilov *et al.* [31]. Briefly, we grew *Shewanella oneidensis* MR-1 strain in anaerobic anodic chambers of original microbial fuel cells. In anodic chamber we used MM synthetic medium with lactate concentration 4 g/l [32]. The electrode material was stainless steel woven mesh (Russian steel grades TU-14- 4-507- 99) produced by Soyuznikhome (Russia). Anodic chamber volume was 250 ml. Cathodic chamber was aerobic. It contained 1x TAE buffer and had volume of 150 ml ml. The chambers were divided by reinforced nafion membrane (membrane thickness 160 µm) produced by Du Pont (USA). The cultivation of bacteria in anaerobic chamber was carried out until the current values reached 30–50 µA from cell (at a voltage of 0.4 V with a load resistance of 8 KΩ). The density of cell culture in the medium reached 2–2.5 g/l.

### Filament extraction

The bacterial fuel cells that produced electrical currents exceeding 30 µA were used for isolation of nanofilaments. These current values fit well the published data [32]. Nanofilaments were extracted in the following way [12], excluding fixation step. Bacterial culture was washed from anode by 10 mmol PBS medium and moved to 50 ml centrifuge tubes.

After that the suspension was vortexed for 5 minutes 4 times with ice cooling in between and onwards centrifuged for 20 minutes at 4°C on 13200 g. The supernatant was collected to another tube, and previous step was repeated for precipitate. Collected supernatant was centrifuged on 150000 g in Optima XPN-100 centrifuge with Type 45 Ti rotor in the sucrose gradient (40%, 20%, 10%, 5%, 2.5%), and 20% fraction with the highest filament concentration was collected. This fraction was additionally centrifuged 2-3 times in Milli-Q water in order to minimize amount of salts and sucrose in suspension.

### Raman spectroscopy

**Fig 1:**
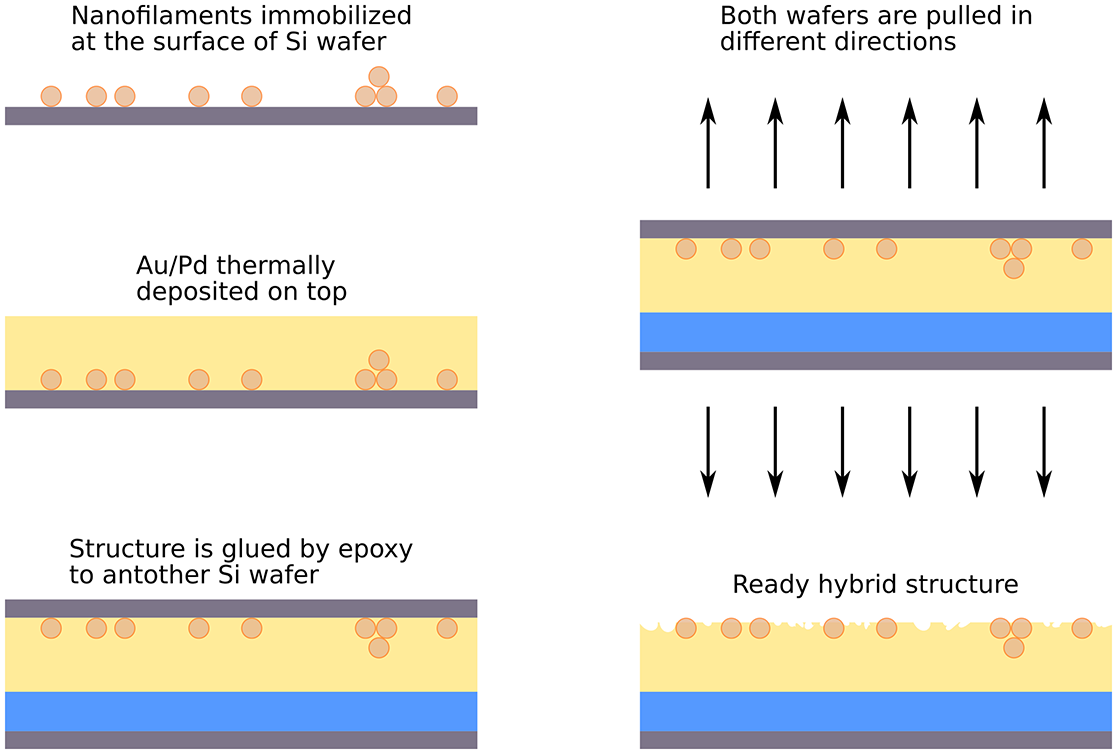
Scheme illustrates the main steps of hybrid structure preparation. Arrows indicate the force applied to detach two wafers. Blue color stands for epoxy layer, yellow for Au/Pd film.

Spectroscopy was preformed on RamMix M532^®^. Excitation wavelength was 532 nm, beam diameter was 2 µm, maximum power was 2 mW. Basically two types of samples were fabricated:hybrid structure (in details below, Fig 1) and a dried drop of the same suspension on a silver colloid substrate for Surface Enhanced Raman Spectroscopy (SERS). Measurements were performed with chaotically moving stage and power of the beam reduced down to 10% to avoid combustion.

#### Hybrid structures

We designed so-called hybrid structures of bacterial nanofilaments embedded into gold-palladium film in order to improve the signal to noise ratio and to avoid sample burn-out. Filaments were immobilized on the surface of silicon dioxide wafer and thoroughly washed. The wafer was glued by the epoxy to the sample holder. After that Au/Pd (80/20% w/w 15–20nm) alloy was thermally deposited by HMNanoFab Stolyarov V.^®^ HV thermal evaporator system on top of the wafer. Layer thickness is tuned to the object height. Another wafer was confidently glued by epoxy to the sample holder. Little drop of epoxy was placed on the top of metal layer and two wafers were pressed to each other. After drying the wafers were detached. Adhesion of the metal layer to the silicon dioxide is weak and the hybrid structure stayed on the epoxy after detachment. The Au/Pd alloy was chosen since it has the smallest reported grain size, so nanofilaments can be clearly observed by atomic force microscope.

### Atomic force microscopy (AFM)

NT-MDT Smena Atomic Force Microscope operating in tapping (non-contact) mode has been used for acquiring all topography data. In almost all cases we utilized MikroMasch cantilevers N15 with resonant frequency of approximately 325 kHz. Further in the text we will determine the diameter of the nanowire identifying it with its cross section height, but not with its width [33]. Such microscope can provide very accurate values for the height of the objects, while its spatial resolution is limited both by the tip radius and the tip form. In general, the observed lateral image is a convolution of the tip’s and object’s form [34].

### Conductivity measurements

#### Nanocontact fabrication for longitudinal conductivity measurements

**Fig 2:**
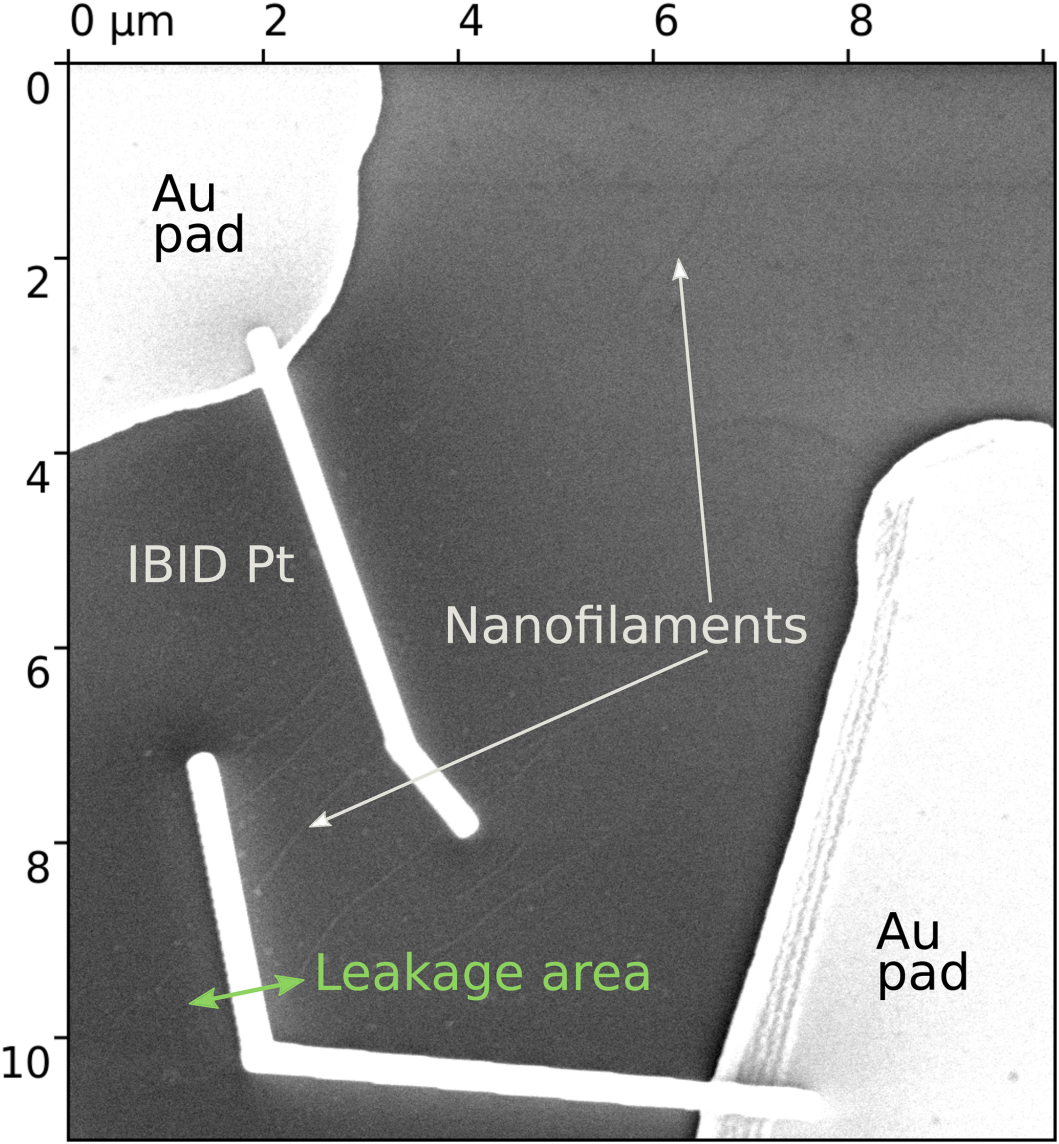
Focused ion beam based fabrication of nanocontacts. Several nanofilaments with organized platinum nanocontacts fabricated by ion beam (marked as IBID Pt on image). The leakage area can be seen as a brighter area around the contact and is indicated by green arrow.

In general, the procedure of contact organization was the same as in Gorby *et al.* [12] We fabricated gold pads on the silicon dioxide surface via photolithography. After that nanofilaments were immobilized on the surface, and, finally, contacts were created by the instrumentality of FIB (see Fig 2). Usually, the size of fabricated nanocontacts was 100 nm width, 100 nm height and 5–12 µm length. However, some important steps of the FIB [35] operation should be highlighted. Gallium beam is highly reactive [36, 37]. Nanocontacts fabricated by electron beam induced deposition (EBID) of metals have low conductivity, 5 orders lower than source metal (Pt in our case). Thus and so it is important to minimize their length in order to reduce contacts contribution to the total resistance in course of two-point measurements (these contacts have small leakage area around them ≈ 300–400 nm [38]). Nanocontacts fabricated by means of gallium beam (*i.e.* ion beam induced deposition—IBID), in contrast, demonstrate high conductivity (1 order lower than pure Pt), but can not be fabricated on the distances shorter than 1 micron. The area around is conductive, and its resistance has close value to resistance of the filament.

#### DC and AC measurements

Due to the studied filaments average length of 3–4 µm and shortcomings of the FIB nanocontact fabrication, all measurements were conducted on two-point devices. DC current was measured via I-V converters with amplification factor 10^8^–10^9^ V/A.

Impedance spectroscopy was carried out on the Z-2000 impedance-spectrometer (Elins, Chernogolovka, Russia). Impedance analysis was performed by ZView-2 (Scribner Association, USA) in a frequency range 1 Hz-2 MHz. The AC (alternating current) voltage amplitude was 50–120 mV. As well as for DC-measurements, two-point regime was applied. Equivalent scheme fitting was performed with ZView-2 (Scribner Association, USA) software.

RH was controlled by humidistats, namely saturated solutions of salts at 19–22°C (partial H_2_O pressure is indicated in parentheses):NaNO_2_ – 66% (11.6 Torr), NaCl – 75% (13.2 Torr), (NH_4_)_2_SO_4_ – 81% (14.2 Torr), KCl – 86% (15.1 Torr), KNO_3_ – 95% (16.7 Torr).

Since water films are formed on the whole surface of silicon wafer covered by suspension (including filaments and contacts), they appear to be electrolyte solutions. Thereby they are a good medium for charge transfer and red-ox reactions on the interface of Pt and Au contacts. Alongside with humidity growth, the total surface of water-metal contact increases. At a certain moment water film covers the upper surface of contacts which leads to sharp change of contribution of reactant diffusion and electrochemical reactions into apparent impedance of the system (see S1 Fig).

## Results

*Shewanella oneidensis* MR-1 has ability to synthesize outer cell appendages with great diversity of structural and functional properties. In our case we analyzed those type of nanofilaments which was the most widespread under chosen conditions of cultivation. The experiments were arranged in the presence of ultra-thin water films with the thickness values controlled by RH. As far as all samples manifested **the same** behavior, we state that charge transfer mechanism in the studied nanofilaments is water-mediated.

### Water mediated charge transfer

Since moisture condensation takes place on both nanofilaments and bare substrate, we compared conductivity data for nanofilament samples and empty circuit test-structures which are presented by nanocontacts fabricated under the same conditions and having the same or very similar geometry on the same substrate as for the sample.

**Fig 3:**
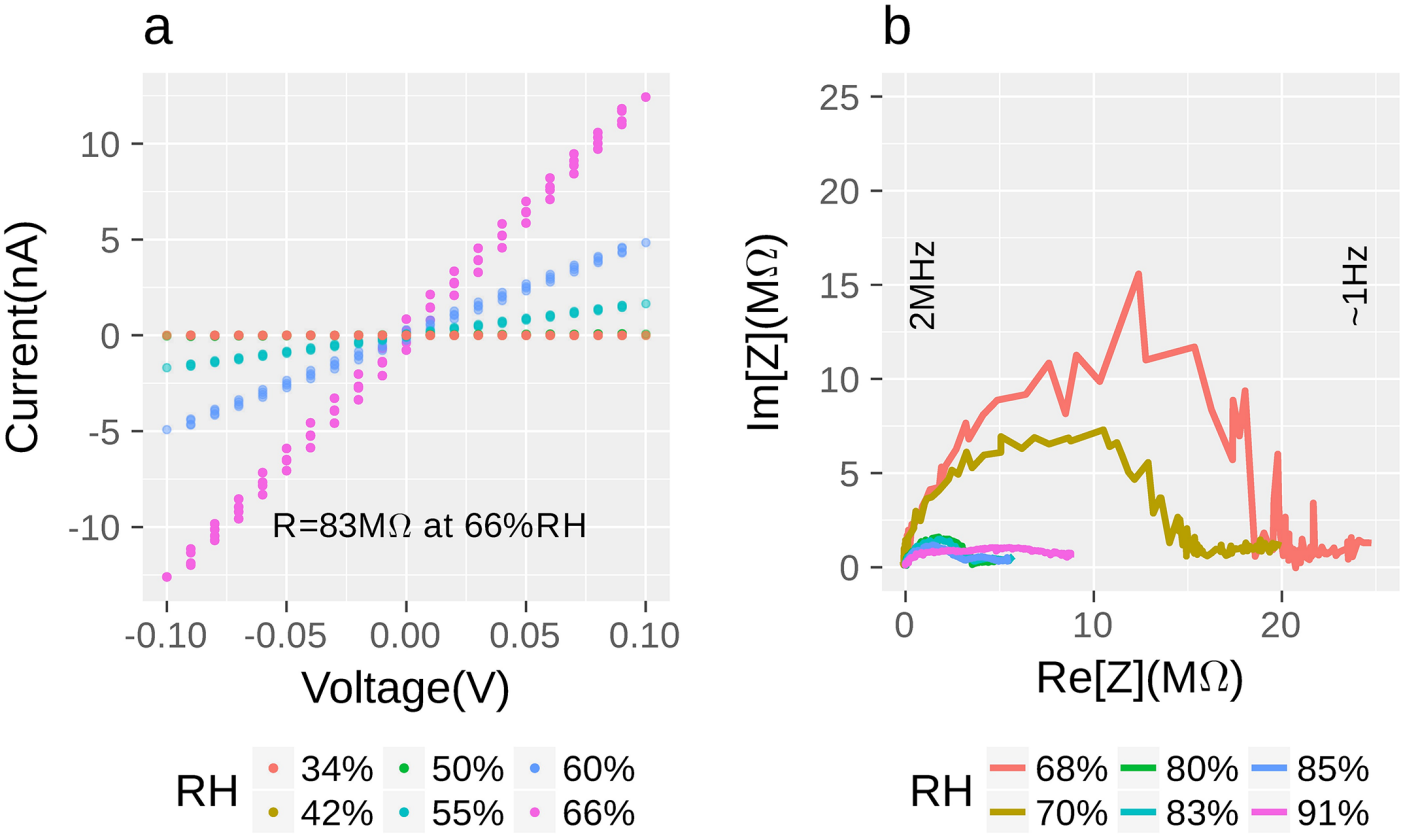
Nanofilament conductance as a function of relative humidity. **a)** DC measurements, voltage sweeping range is −100–100 mV **b)** AC-measurements, Nyquist plot, frequency range is 1 Hz-2 MHz. Relative humidity (see legend) was controlled by humidistats (see Materials and Methods). It should be denoted that after reaching critical humidity level (≈90%) the impedance changes its shape radically (purple line), semicircles become indistinguishable.

Both the sample(see Fig 3a) and the empty circuit test-structure (see S2 Fig) yielded qualitatively similar I-V curves (DC) with negligible quantitative difference at **low voltage**. The difference increased at voltages above 0.5 V, however electrochemical reactions, possible under these conditions, limit the measurements.

**Fig 4:**
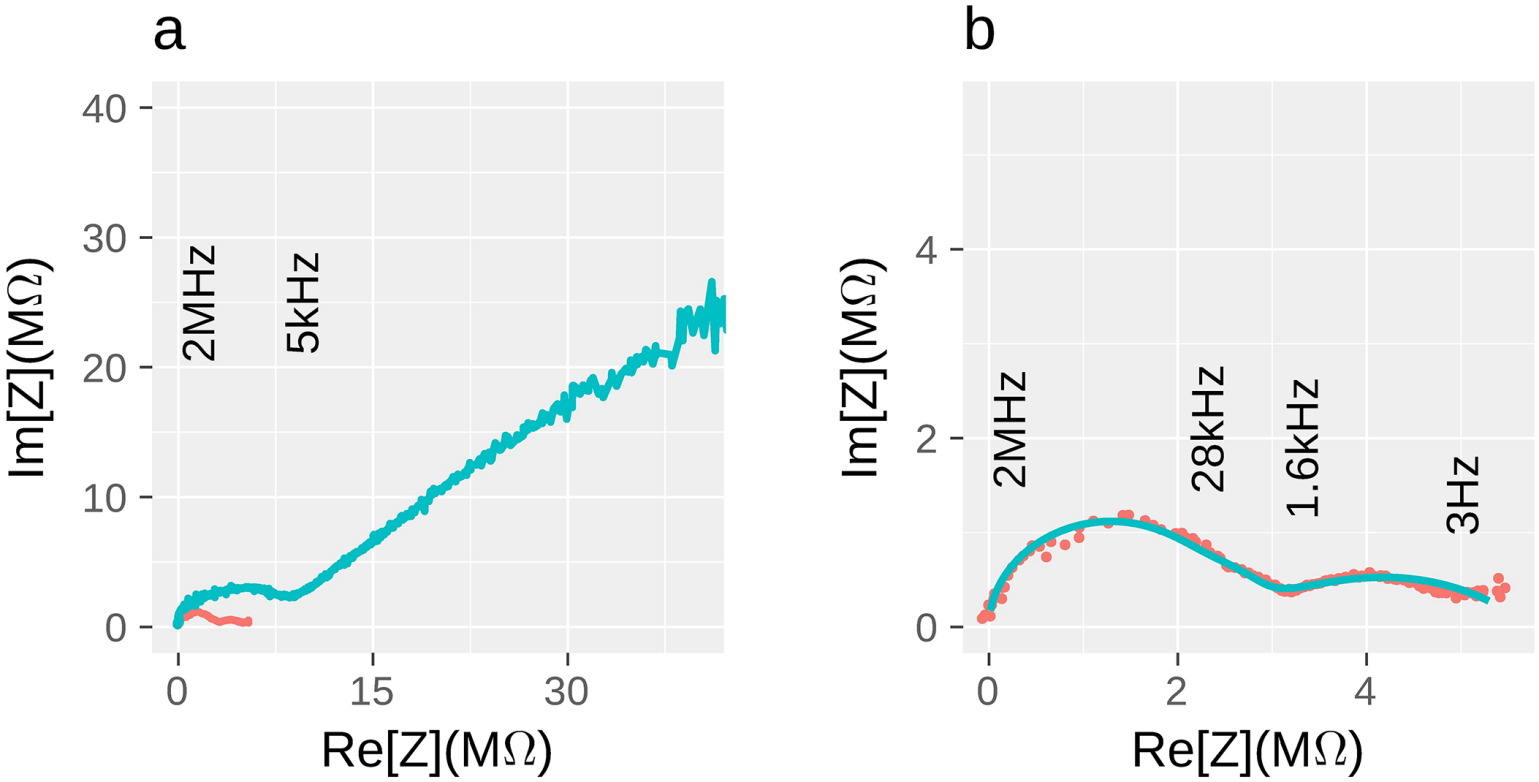
Impedance Nyquist plots. **a)** Comparison of the filament (red line) and empty circuit with almost identical nanocontact geometry (blue line); half circle that continued by straight line with the slope of *ca.* 45° represents reactant diffusion (Warburg element) in test-structure, while nanofilament hodograph consists only of several half-circles. However, the straight line feature for the nanofilament sample can be found in lower frequency region. Frequencies demonstrated on this figure correspond only to the blue line. Full frequency range for all measurements is 1 Hz–2 MHz. Both impedances were acquired at 80% relative humidity. **b)** Characteristic nanofilament hodograph: experimental data (red dots) and calculated line for equivalent scheme (blue line).

Further investigation was based on impedance spectroscopy (IS). Im(Z) vs. Re(Z) (Nyquist plot, imaginary part of impedance (Z) versus real) dependencies are specific for various objects and [39] this fact can be helpful for understanding the origin of charge transport. For AC (alternating current) measurements 120 mV voltage amplitude was applied. Nyquist Plots in the frequency range 1 Hz-2 MHz of impedances at various RH are presented on Fig 3b. Qualitative and quantitative differences of nanowire’s impedance for lower and higher humidities are demonstrated on Fig 3b and the distinction between the sample and test structure is shown on Fig 4a.

**Fig 5:**
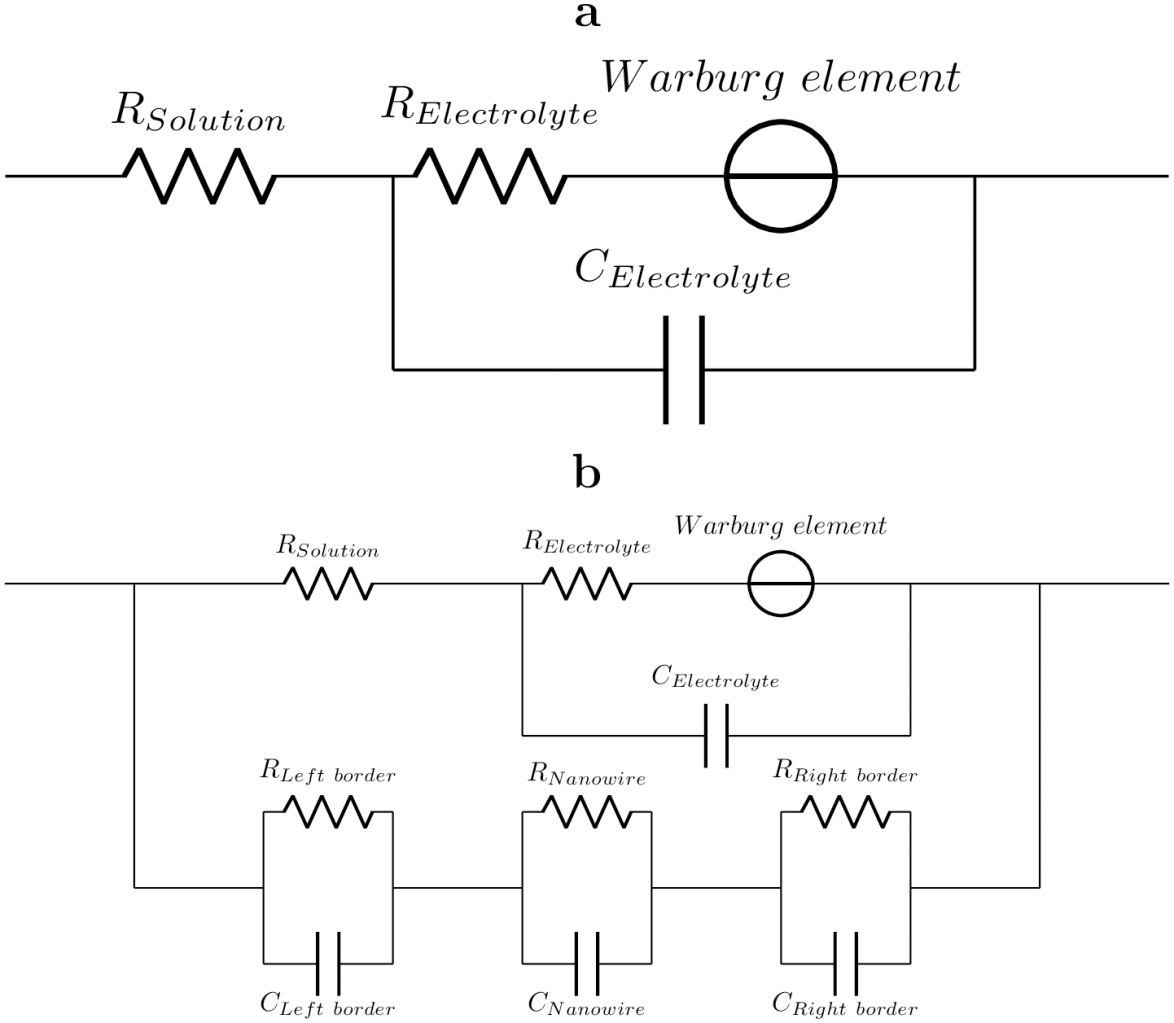
Equivalent schemes. **a) Scheme ES (empty-structure)** Equivalent scheme for empty circuit test-structure, that model red-ox reactions and diffusion of reactants in surrounding medium. Technically, this scheme models electrolyte film impedance. **b) Scheme ES+NF (empty-structure+nanofilament)** This scheme describes the sample with nanofilament (three bottom R-C blocks, two for electrochemical reactions occurring on the left and right nanowire-contact interfaces and one for the nanofilament itself) in parallel with the electrolyte film impedance. This scheme works at all humidities.

The empty circuit test-structure (see Fig 4a) can be described by the classic Warburg element [40] ${\text{Z}}_{\text{W}}=\frac{{\text{A}}_{\text{W}}}{\sqrt{\omega }}+\frac{{\text{A}}_{\text{W}}}{i\sqrt{\omega }}$ (linear part of the plot) at low frequencies (below 5 kHz), which generally corresponds to reactant diffusion in electrolyte solution. To determine the parameters of red-ox reactions and diffusion of reactants in surrounding medium we fitted impedance of empty circuit test-structure by **scheme ES (empty structure)** (see Fig 5a).

Onward the sample with nanofilament can be presented by combination of empty circuit in parallel with three R-C blocks, one for nanofilament and two for electrochemical reactions occurring at the left and right nanofilament-contact interfaces (see **scheme ES+NF** Fig 5b). It simplifies the contribution of the nanofilament into the apparent impedance but manages to operate at any RH levels. One also can model the nanofilament by transmission line element and obtain higher fitting accuracy in low-frequency region, sacrificing high RH levels. However the numerical values obtained from the fitting calculations may be ambiguous, while the qualitative dependencies on the external parameters are much more informative. We failed to find linear feature for nanowire, at least in available frequency range. Such behavior points to the presence of non-diffusive process in nanofilament charge transfer mechanism.

Characteristic resistances (see Table 1) of surrounding media calculated for empty circuit modeled by **scheme ES** (R_electroyle_ and Re(Z_Warburg element_)) appear to be ≈ 0.1–1 GΩ and coincide with the values obtained from a nanowire-electrolyte system impedance fitted by **scheme ES+NF**.

**Table 1:** Characteristic parameters of equivalent scheme ES + NF.

Parameter	low RH (≈60%)	high RH (≈90%)
Re(Z_Warburg element_) [MΩ]	600	200
R_electrolyte_ [MΩ]	100	20
R_nanofilament_ [MΩ] for 4 µm	0.5-5	0.5-5
R_right/left border reactions_ [MΩ]	10-20	0.5
C_electrolyte_ [µF/cm^2^]	≈10	≈100
C_right/left border reactions_ [µF/cm^2^]	≈1	≈10

**Fig 6:**
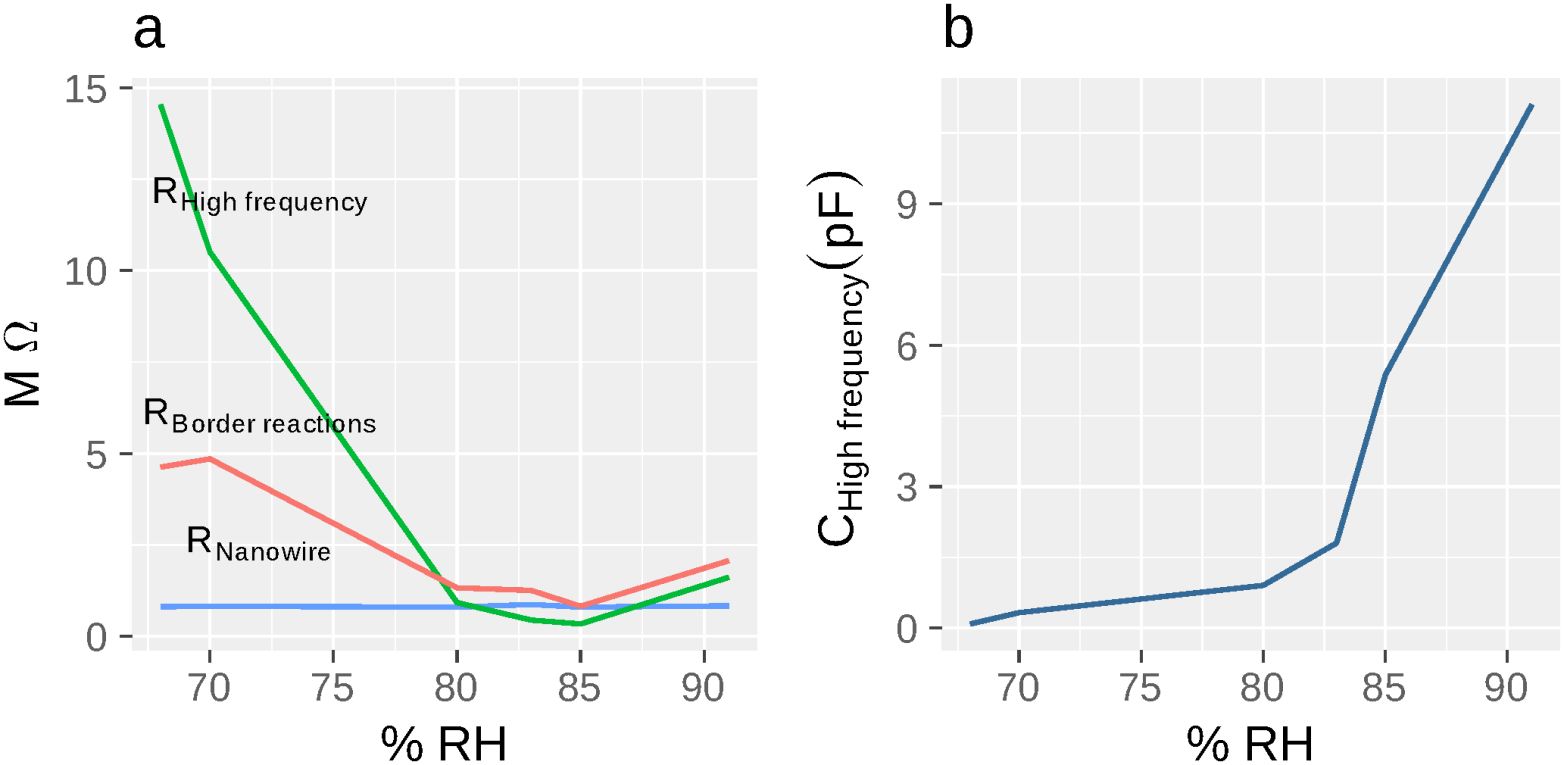
The dependence calculated parameters on water content. **a** Characteristic dependence of active parameters fitted by **Scheme EF** on RH. R_High frequency_ and R_Border reactions_ decrease with increasing water content. R_nanofilament_ almost doesn’t depend on the water content, once the resolution is enough to distinguish all half-circles in hodograph. We assume this parameter to represent resistance of nanofilamet. **b**) High frequency capacitance calculated by **Scheme EF** C_High frequency_ increases alongside with humidity. Its numeric values, according to the size of fabricated nanocontacts, fit its characteristic value.

As it has been stated above that equivalent schemes provide some apparent values. But the quantitative value of modeled parameters can be ambiguous. The dependence of certain equivalent scheme parameters on external factor (RH in our case) is more informative. There are several resistances that decrease (see Fig 6a, and Table 1) alongside with growing humidity (R_electrolyte_, Re(Z_Warburg element_), R_border reactions_ in **scheme ES+NF**), and the only one parameter, R_nanofilament_ that is almost independent on RH.

C_Border reactions_ and C_electrolyte/High frequnecy_ (see Fig 6b) depend on RH (both values grow slowly with the smooth change of nanocontacts surface, that is covered) until the break (see S1 Fig for explanation of surface sharp increase) and fit its characteristic values (100 and 10 µF/cm^2^—respectively [39], see Table 1) assuming that interaction occurs on the whole length of Pt contacts (several microns length, ≈ 100 nm wide, ≈ 100 nm height).

### Raman spectroscopy

**Fig 7:**
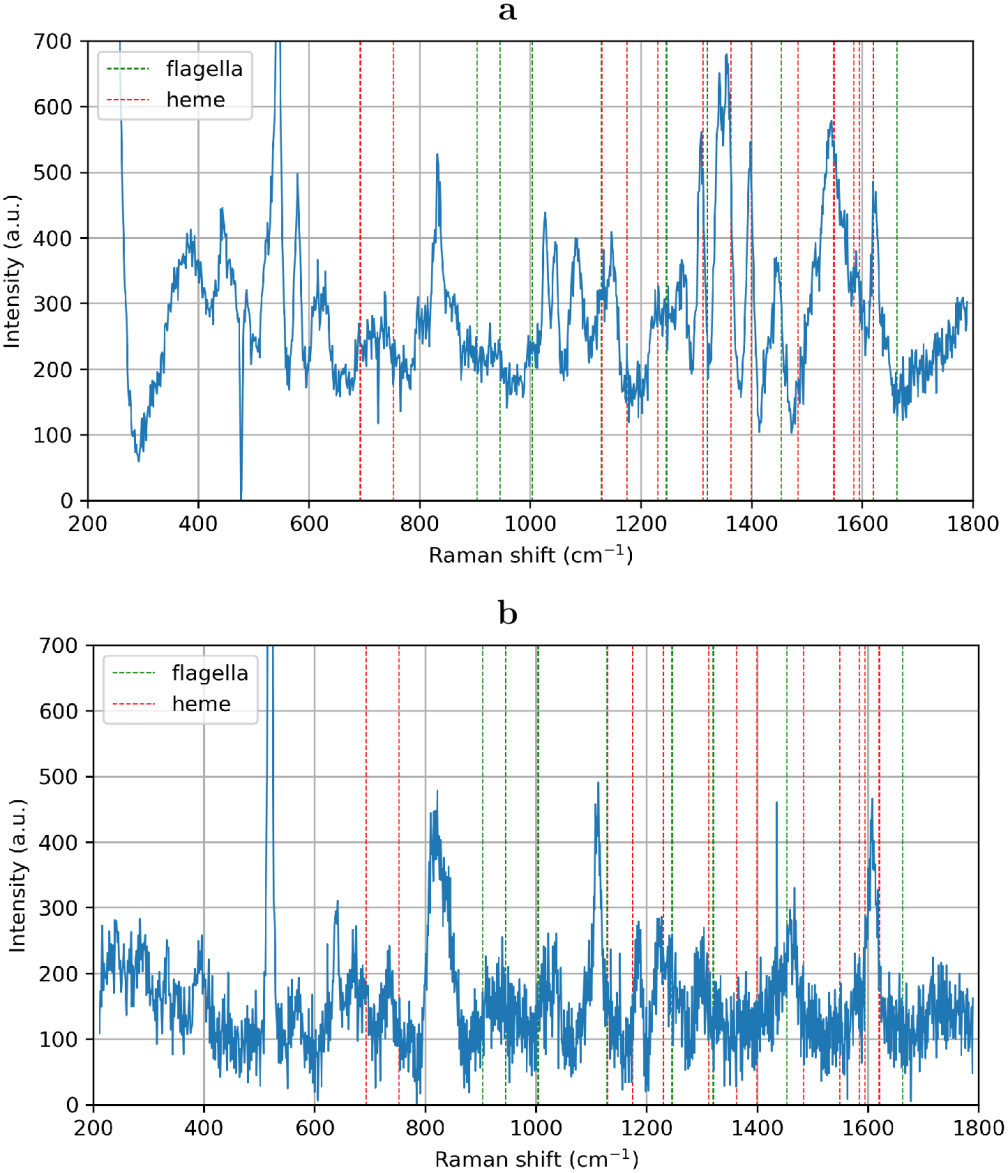
Raman spectroscopy. Surface Enhanced Raman Spectrum (SERS) of nanowires immobilized on the surface of silver colloid substrate and Raman spectra of nanowires embedded in metal film. Dashed red lines correspond to the perfectly matching cytochrome spectral lines. Dashed green lines correspond to flagellin spectrum. Tabulated data and comparison with earlier published results of Raman spectroscopy of bacterial colonies and various bacterial proteins are presented in S1 Table.

We applied Raman spectroscopy (see Fig 7) to characterize chemical composition of nanfilaments. To avoid artifacts resulting from nanoobjects combustion we used Surface Enhanced Raman Spectroscopy (SERS) on silver colloid substrate (Fig 7a) and filaments embedded in metal film (Fig 7b) (see Materials and Methods). According to the comparison of SERS data obtained on our samples and published materials (see S1 Table) we can affirm that the isolated suspension contains six-coordinated Fe^II+^ in the intermediate spin state [41] and quinones in various red-ox states. These data coincide with previously reported Raman spectra of decaheme cytochromes (MtrC and/or OmcA [42]) associated with periplasmic membrane [4, 43] and spectral signatures related to the cell surface [42, 44, 45]. Isolated suspension does not contain any signatures of flagella or flagellin [46] which could be distinguished from heme lines. Moreover, one can not observe any line of very strong intensity in spectra.

### Atomic-force microscopy (AFM)

#### Morphology

**Fig 8:**
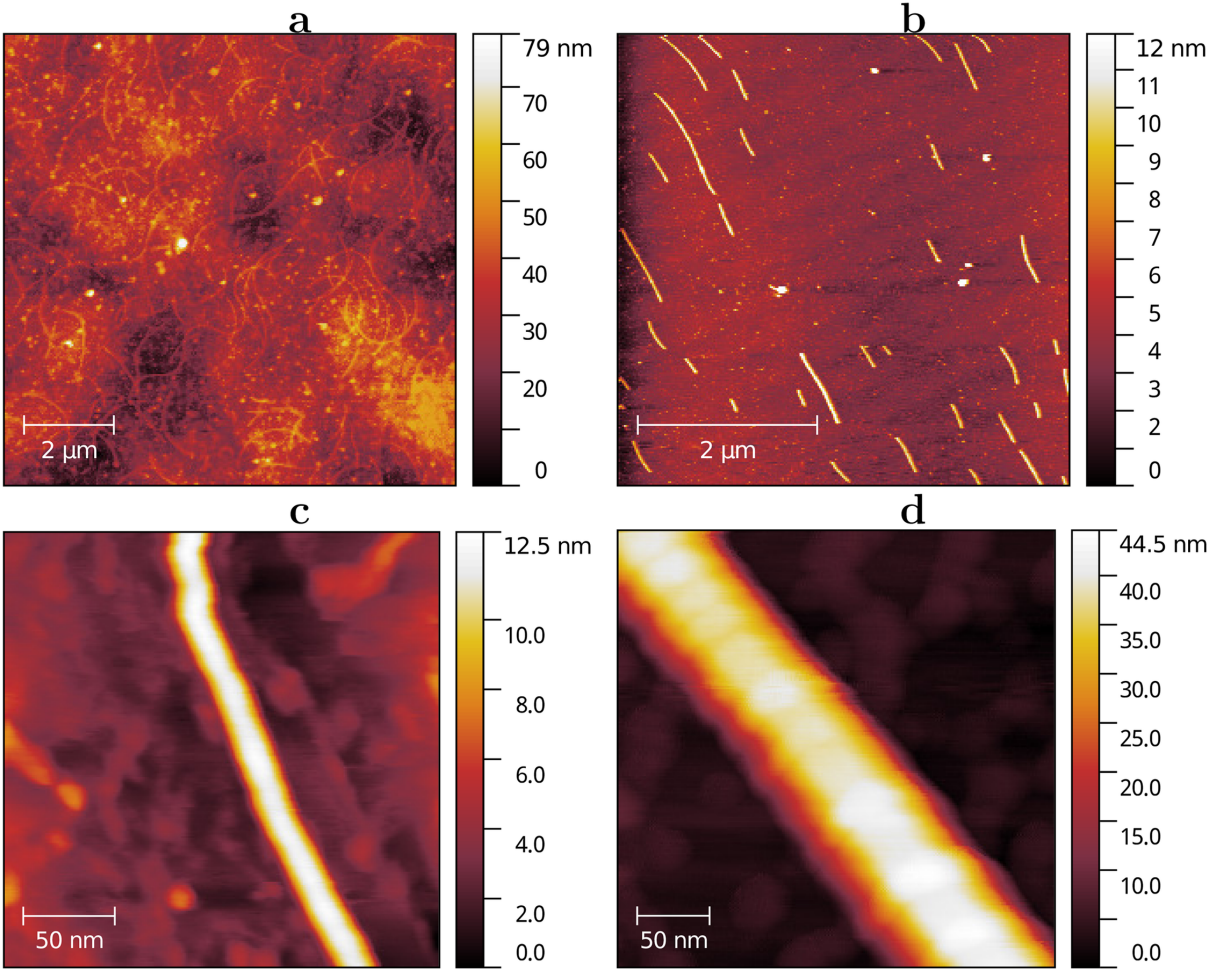
AFM morphology of *Shewanella* filaments. **a** A drop of the nanowire suspension was put on the surface of the silicon dioxide and ten minutes later it was blown away. After deposition many filaments demonstrated wavy form. However, in the case of pneumo-spray deposition **b** all the nanowires were straight. The average diameter was measured to be 7 ± 2 nm determined by the nanowire cross-section height. This is due to much lower lateral resolution in comparison to transverse (see Materials and Methods). **c** Close up view on the nanowire. No “submorphology” can be observed. **d** Composite nanofilament with a diameter of approximately 45 nm is rarely presented in the suspension.

In accordance with spectral measurements, nanofilaments or at least suspension with nanofilaments contain disparagingly small quantity of flagellin. Furthermore we performed morphological analysis on the large quantity of sample to study the structure of presented wires. We observed that most of the nanofilaments are 7 ± 2 nm and obtain different forms that are mainly governed by deposition conditions rather than by the nanofilament origin. If the nanofilaments had been deposited from the drop of the solution, many of them would have wavy form (see Fig 8a), while more aggressive deposition conditions like pneumo-spray, would have resulted only in straight, but shorter filaments (see Fig 8b). At the same time, appreciable difference in morphology and size was captured in case of filament structures rarely observable after any deposition (see on Fig 8d). These filaments are composite and have a sub-morphology of twisted wire.

Combining morphological data and Raman spectroscopy we can state that we studied conductive nanowires produced by *Shewanella Oneidensis* MR-1 at the certain cultivation conditions, that clearly contain large amount of six-coordinated iron atoms. Due to the lack of conductivity at ambient conditions we can not state that these nanofilaments are of the same nature as in Pirbadian et al. [4]

## Discussion and conclusion

The resistance of nanofilament R_nanowire_ component which has no dependence on RH in all measured ranges can be attributed to both electron and proton transport through single aqueous layer. Unfortunately, current level of technique permits to extract proton conductivity by means of transient current measurements. However, the known successful examples of application of this methodology are related to the samples with characteristic dimensions of hundreds of microns. Our samples had only hundreds of nanometers length, *i.e.* we could not measure the current decay process and determine its temporal constants by means of affordable instruments.

At the same time we can see that the observed values of conductivity at n.c. of our samples are at least two orders of magnitude lower than the known results of El-Naggar lab [10, 12] and several orders higher than the values obtained for nanofilaments constructed from artificial thiophene-enriched peptides with approved electron component of conductivity. The measurements of Ashkenasy lab [24, 26, 27, 47] were conducted with thoroughly deionized materials without special fixation procedures. The resistance of the filament R_nanofilament_, as estimated above, is of an order of 1 MΩ for 1 µm length at 80–85% relative humidity.

Electron hopping mechanism was previously suggested as hypothetical mechanism of electron conductivity in bacterial **nanowires** of *Shewanella oneidensis* MR-1 [48]. Each iron atom in cytochrome species represents the center for electron localization. Conductivity in this model is controlled by overlap of electron wave-functions. The distance between two neighboring hemes r_nn_ is crucial for conductivity: closer localization results in higher conductivity. According to this model the resistance of the studied nanofilaments should decrease sharply in the presence of water films. It qualitatively coincides with the measured dependencies, although the precision of our equipment does not allow to track this effect accurately at low RH levels due to high resistances.

The value of observed decrease of resistance is too strong to be explained by the change of interheme distance [9]. In electron hopping model: *R* ∝ 1/(*n*|*H*|^2^), where *n* is the charge hopping cites concentration and *H* is the overlap integral. We can roughly write, *R* ∝ *r*_*nn*_ ⋅ exp(2*βr*_*nn*_), where *β* [8, 49–52] is of an order of 1 Å^−1^ for various peptide systems. It leads to obviously nonphysical result of 10 nm interheme distance change due to the presence of water.

Water molecules may have an effect on both types of conductivity, ionic and electron. The ability of ions to diffuse in medium is limited by interaction with various charged and polarized structures in bacterial filaments. Increase of water concentration leads to formation of bulk aqueous phases with higher diffusion constants for all non-aqueous ions. On the other hand protons and hydroxyl anions may participate in charge doping of various aromatic systems presented in proteins. These interactions modify the band structure of aromatics and accordingly change electron transfer constants. Also, water is needed to move equilibrium in reactions of formation/deformation of semiquinones from quinones and quinoles to the right thus supporting higher concentration of aromatic anion-radicals [28]. MQ-7 quinone and riboflavin are natural components of *S. oneidensis* extracellular matrix [53–55]. The presence of quinones in studied samples is supported by Raman scattering. In our case the semiconductor/dielectric bi-exponential model previously utilized for various amorphous materials of biological origin including melanin [28] gives the most prominent result. The drastic change of dielectric permittivity of the sample due to hydration seems to be more natural than 10 nm change of interheme distances provided by hopping model.

Limitations of our measurements technique does not allow us to detect conductivity below RH 45%. However, we can correlate the obtained R_nanofilament_ dependence on RH with predictions of the model of amorphous semiconductor in the range, where the level of conductivity is almost saturated.

$$\sigma =\sigma_{0}\cdot \exp(-\frac{E_{D}}{2RT})\cdot \exp\left\{\frac{e^{2}}{2RTr}(\frac{1}{\kappa }-\frac{1}{\kappa^{\prime }})\right\}\left[56,57\right],$$

where *σ* is the conductivity, *σ*_0_ is conductivity in a dry state, *E*_*D*_ – “band” gap of the material, *T* is temperature, *r* is screening length, *κ* and *κ*′ are dielectric constants in a dry state and at the relevant hydration respectively. At the same time, R_High frequency_ dependence on RH can be interpreted with the help of “semiquinone model” [28] which contains two components. Firstly, the increasing concentration of hydroxonium cations which may give contribution to ion conductivity. Secondly, the increasing concentration of semiquinone anion-radicals contributes to both electron and ion conductivity in the sample.

Finally, precise control of environment during measurements has revealed peculiar properties of charge transfer in studied nanofilaments of *Shewanella oneidensis* MR-1. We demonstrate, that application of impedance spectroscopy provide more information about conductivity mechanisms in microbial nanofilaments than traditional DC-measurements. Basing on the previous studies of melanin and artificial self-assembling peptides, which have notable similarities with the studied object, we state that one of the types of nanofilaments intensively produced by *Shewanella oneidensis* MR-1 under discussed conditions, exhibit complex type of conjugated ion-electron charge transfer.

## Supporting information

S1 Fig Scheme illustrating the change of water-metal surface with increasing humidity. At each particular RH level, there is a water layer lying between IBID Pt contacts above the bacterial nanofilament. Having reached a certain critical RH value, the thickness of the water layer overcomes 100–150 nm contact height and 300 nm of Au pads. At low humidity levels, below 30%, even the nanowire is not completely covered.
(TIFF)

S2 Fig Empty-circuit test structure DC I-V curves in dependence on RH. Comparing to Fig 3a no perceptible difference can be observed.
(TIFF)

S1 Table Tabulated Raman spectroscopy data. (PDF)
