# Supplementary figures and images for: Impedance spectroscopy of single bacterial nanofilament reveals water-mediated charge transfer
Source: PLoS One. 2018 Jan 19;13(1):e0191289. doi: 10.1371/journal.pone.0191289 (PMC5774759; doi:10.1371/journal.pone.0191289)

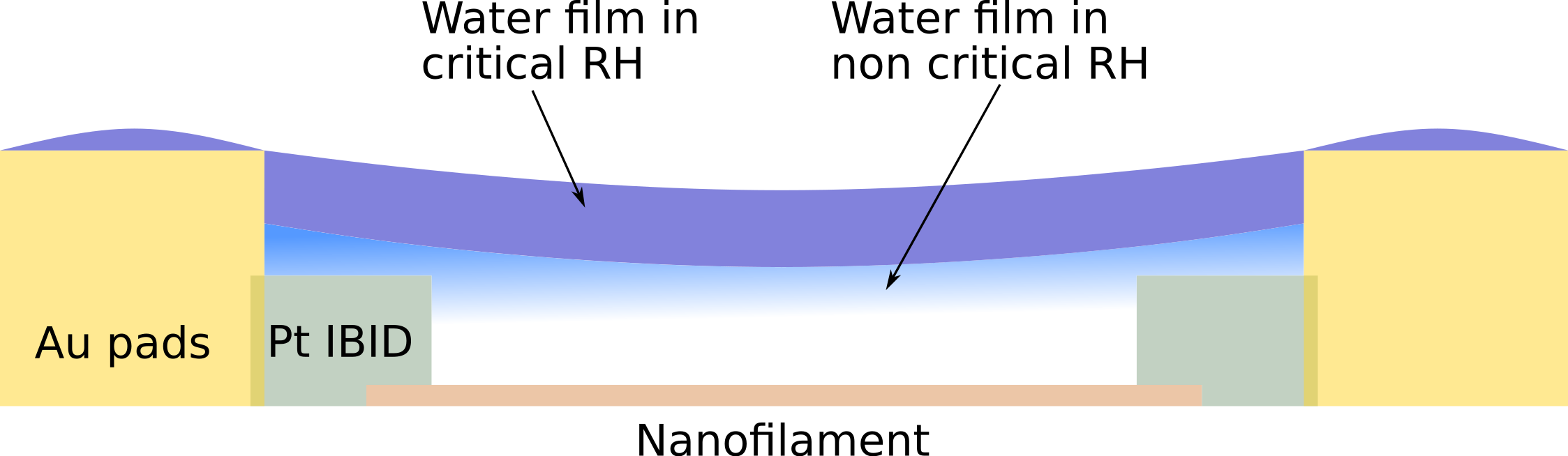

Supplement: S1 Fig — At each particular RH level, there is a water layer lying between IBID Pt contacts above the bacterial nanofilament. Having reached a certain critical RH value, the thickness of the water layer overcomes 100–150 nm contact height and 300 nm of Au pads. At low humidity levels, below 30%, even the nanowire is not completely covered. (TIFF) [file pone.0191289.s001.tiff]

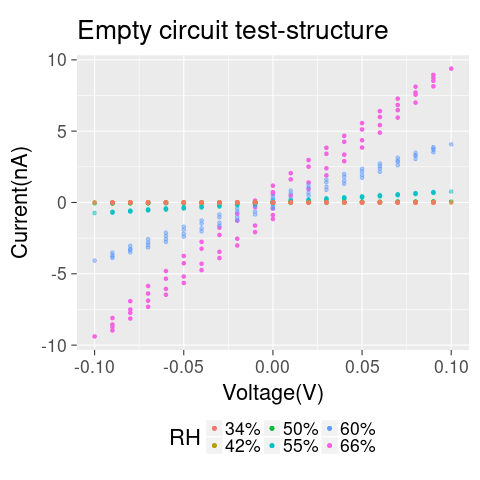

Supplement: S2 Fig — Comparing to Fig 3a no perceptible difference can be observed. (TIFF) [file pone.0191289.s002.tiff]
