# Supplementary material for: Impedance spectroscopy of single bacterial nanofilament reveals water-mediated charge transfer
Source: PLoS One. 2018 Jan 19;13(1):e0191289. doi: 10.1371/journal.pone.0191289 (PMC5774759; doi:10.1371/journal.pone.0191289)
Supplement: S1 Table — (PDF) [file pone.0191289.s003.pdf]

Table S1. Tabulated Raman Spectroscopy data and comparison with published materials

Experimental Data				Shewanella and heme lines										Flagella and Flagellin				Comment
SERS		HS Raman		Porfirine[1]		MtrC[2]		OmcA[2]		CS[2]		CS II[3]		Flagella[4]		Flagellin[4]		
		170	w															No comment
		216	vw															Histidine
232	vs	238	w															γ_{24} ImH[5]
		268	vw															No comment
		284	vw															No comment
		332	vw															No comment
												346	w					No comment
**386**	m	**394**	m									394	vs					Indicates six-coordinated Fe^{II} in low-spin state[6]
443	m	443	vw									447	m					γ_{22} ImH [5]
												466	m					No comment
487	w																	No comment
500	vw											500	m					No comment
		518	vs															SiO_2 substrate
525	w																	No comment
												534	m					No comment
543	s																	Substrate
579	s	571	w															No comment
615	m																	No comment
630	m	638	m									630	vs					No comment
666	w	668	vw															No comment
												675	m					No comment
690	vw	690	w	692	m													Heme
												712	w					No comment
738	m	735	m															No comment
				752	s									750	w			No comment
777	vw	778	w									768	m					No comment
802	w											808	w					No comment
832	s	827	s															CCH (aliphatic)[7]
860	w											864	s					No comment
														903	m	903	w	Flagellin(no match)
														940	m	945	w	Flagellin(no match)
1000	vw											998	vs	1005	w	1003	w	No comment
1026	m	1018	w															No comment
1045	m	1036	w									1036	m					No comment
												1068	s					No comment
1081	s																	No comment
		1112	s															No comment
1129	m			1129	s							1127	m	1127	m	1127	w	Due to low intensities and possible line shit[8] there is no prove of flagellin presence
1146	m	1147	w															No comment
1174	vw	1185	m	1174	m													Rocking of Hs on C_β atoms[1] and $C_\beta - C_\phi$ bond stretching[9]
1227	m	1224	m	1230	m	1227	w	1234	w	1224	s	1238	s					Rocking of Hs on N atoms[1]

Experimental Data				Shewanella and heme lines										Flagella and Flagellin				Comment
SERS		HS Raman		Porfirine[1]		MtrC[2]		OmcA[2]		CS[2]		CS II[3]		Flagella[4]		Flagellin[4]		
1250	w	1243	w			1252	w	1254	w	1246	s			1248	s	1246	s	3° amide [7]. Although there is a match with both flagella and flagellin, lines are indistinguishable with pure decaheme lines. Intensities diff. too much
1275	m					1279	vw	1271	vw			1269	s					
								1287	vw	1288	vw							No comment
1307	s	1303	s	1312	s	1316	vs	1314	vs	1306	vs	1307	vs					$N-C_\alpha-C_\beta$ or $HN-C_{\alpha'}-C_{\beta'}$ assym. stretching[1] and ν_{21} ImH [5]
														1327	m	1322	m	Flagellin (no match)
1341	vs	1336	w															Peak in multiple wide line
**1356**	vs	**1357**	vw	1362	s	1373	m	1368	m	1362	s							ν_4 ImH[5]. Also indicates six-coordinated Fe^{II} in low-spin state[6]
1398	vs	1407	w	1399	vs	1399	s	1399	s	1398	vs							$C_\alpha-N(H)-C_\alpha$ sym. stretching[1] and ν_{29} ImH[5]
1432	m	1435	m			1430	m					1425	m					Match with spectra and flavine (semiquinone)[9]
1445	m	1459	m			1458	vw	1446	w	1448	m			1452	vs	1453	vs	CH_2 scissoring[7]. Although there is a match with both flagella and flagellin, lines are indistinguishable with pure decaheme lines. Intensities diff. too much
1463	w	1467	m							1466	s							No comment
1514	m	***1503***	vw	1483	s	1500	vw	1500	s	1494	w							$C_\alpha-NH-C_\alpha$ sym. stretching[1] Also indicates Intermediate-Spin Fe^{II}[10]
**1541**	vs	**1547**	w	1548	vs			1536	m	1528	m							ν_{11} ImH[5]. Also indicates six-coordinated Fe^{II} in low-spin state[6]
1569	m	***1561***	w			1564	vs		m									ν_{38} ImH[5] Also indicates Intermediate-Spin Fe^{II}[10]
1589	m	***1605***	vs	1584	vs	1588	vs	1588	vs	1594	vs	1583	vs					ν_{37} and ν_2 ImH[5] Also indicates Intermediate-Spin Fe^{II}[10]
1621	s	1617	m	1620	vs					1617	vs							ν_{10} ImH[5] also may be a trace of semiquinone[9]
1642	m					1639	vs	1641	s									MtrC and OmcA match. ν_{10} ImH[5]. Also indicates Intermediate-Spin Fe^{II}[10]
												1660	m	1652	vs	1662	vs	Flagellin in aerobic cells

Intensities: **vw** - very weak, **w** - weak, **m** - medium, **s** - strong, **vs** - very strong. Other: **ImH** - Imidazole complex of cytochrome. γ_i or ν_i - lines numbering according to given citations. Numbers in **bold** indicate the most important lines in spectra.

References

1. Aydin M (2013) DFT and Raman spectroscopy of porphyrin derivatives: Tetraphenylporphine (TPP). *Vibrational Spectroscopy* 68:141–152.
2. Wang Y et al. (2013) Single-cell imaging and spectroscopic analyses of Cr(VI) reduction on the surface of bacterial cells. *Langmuir : the ACS journal of surfaces and colloids* 29(3):950–6.
3. Ravindranath SP, Henne KL, Thompson DK, Irudayaraj J (2011) Surface-enhanced Raman imaging of intracellular bioreduction of chromate in Shewanella oneidensis. *PLoS ONE* 6(2):1–10.
4. Uchiyama T et al. (2008) Raman optical activity of flagellar filaments of Salmonella: Unusually intense ROA from L-type self-assembled protein filaments and their possible higher level chiral organization. *Vibrational Spectroscopy* 48(1):65–68.
5. Othman S, Le Lirzin A, Desbois A (1994) Resonance Raman investigation of imidazole and imidazolate complexes of microperoxidase: Characterization of the bis(histidine) axial ligation in c- type cytochromes. *Biochemistry* 33(51):15437–15448.
6. Linard JE, Shriver DF, Basolo F (1980) Structure-sensitive resonance Raman bands of capped tetraphenylporphyrinatoiron complexes. *Proc.Natl.Acad.Sci.U.S.A* 77(0027-8424 (Print)):1741–1744.
7. Preciado-Flores S et al. (2011) SERS spectroscopy and SERS imaging of Shewanella oneidensis using silver nanoparticles and nanowires. *Chemical communications (Cambridge, England)* 47(14):4129–4131.
8. Ghosh C, Mukherjee S, Dey SG (2013) Direct electron transfer between Cyt c and heme-Aβ relevant to Alzheimer's disease. *Chemical communications (Cambridge, England)* 49(51):5754–6.
9. Tripathi GNR (1981) Resonance Raman scattering of semiquinone radical anions. *The Journal of Chemical Physics* 74(11):6044–6049.
10. Spiro TG, Burke JM (1976) Protein control of porphyrin conformation. Comparison of resonance Raman spectra of heme proteins with mesoporphyrin IX analogues. *Journal of the American Chemical Society* 98(18):5482–9.
